# The Protective Effect of Evodiamine in Osteoarthritis: An *In Vitro* and *In Vivo* Study in Mice Model

**DOI:** 10.3389/fphar.2022.899108

**Published:** 2022-06-20

**Authors:** Shuyuan Xian, Zeng Lin, Chao Zhou, Xing Wu

**Affiliations:** ^1^ Department of Orthopedics, Shanghai Tenth People’s Hospital, School of Medicine, Tongji University, Shanghai, China; ^2^ Department of Orthopaedics, Yinshanhu Hospital of Wuzhong District, Suzhou, China

**Keywords:** osteoarthritis, NF-κB, evodiamine, potential agent, anti-inflammation

## Abstract

Osteoarthritis (OA) is a chronic disease with high economic burden characterized by cartilage degradation and joint inflammation. Evodiamine (EV), which can be extracted from *Evodia rutaecarpa* (Rutaceae), is a traditional Chinese medicine to treat inflammation, cardiovascular disorders, infection, and obesity. Studies have shown that EV can suppress the activation of immune cells and restrain the secretion of pro-inflammatory cytokines. However, it is still not well known about its role in the treatment of OA. In this study, we utilized interleukin-1β (IL-1β)–stimulated mouse chondrocytes *in vitro* and the destabilization of the medial meniscus (DMM) model *in vivo* to demonstrate the anti-inflammatory properties of EV in OA. The results suggested that EV decreased the generation of NO, IL-6, TNF-α, and PGE2. Meanwhile, the increased expression of iNOS, COX-2, and MMP-13 and the degradation of aggrecan and Col-II were significantly alleviated by EV in IL-1β–activated mouse chondrocytes. Moreover, EV can inhibit the considerable IL-1β–stimulated phosphorylation of the NF-κB signaling pathway and nuclear translocation of p65, compared with the control group. Furthermore, EV alleviated cartilage degeneration and reversed the increased Osteoarthritis Research Society International (OARSI) scores in the OA model *in vivo*. Our study demonstrates that EV can suppress inflammation *in vitro* and cartilage degeneration *in vivo* in OA, which implies that EV may be a potential candidate for the treatment of OA.

## Introduction

Osteoarthritis (OA), a chronic and progressive joint disease characterized by the proliferation of subchondral bone and deterioration of articular cartilage, can cause joint dysfunction, seriously degrade the quality of life, and burden the economy on a global level (Glyn-Jones et al., 2015). With the aggravation of population aging, the number of patients with osteoarthritis has dramatically increased (Hinton et al., 2002). Age, gender, trauma, obesity, genetics, inflammation, and living conditions are the risk factors for OA (Egloff et al., 2014; Wallace et al., 2017). Numerous studies suggest that inflammatory cytokines and local inflammatory responses play vital roles in the pathogenesis and progression of OA (Kapoor et al., 2011). Specifically, interleukin-1β (IL-1β) is one of the key factors involved in OA development, which can aggravate inflammation response by inducing prostaglandin E2 (PGE2), cyclooxygenase-2 (COX-2), inducible nitric oxide synthase (iNOS), matrix metalloproteinase-13 (MMP-13), and thrombospondin motifs (ADMATs) in chondrocytes (Goldring et al., 1994; Bondeson et al., 2006; Jenei-Lanzl et al., 2019). Furthermore, IL-6 and TNF-α levels are abnormally upregulated in cartilage, synovial fluid, and the synovial membrane of OA patients, implying that they play crucial roles in the pathogenesis of OA (de Lange-Brokaar et al., 2012). Therefore, blocking the IL-1β–activated inflammation response might be an effective means to slow or prevent OA progression.

Nuclear factor κB (NF-κB), a dimer transcription factor, participates in the development of OA through regulating gene transcription involved in the inflammatory response, immune response, and cell differentiation. Numerous studies have found that pro-inflammatory factors IL-1β, TNF-α, and LPS can activate the NF-κB signaling pathway and intensify p65 phosphorylation and nuclear translocation, which promote the transcription and translation of target genes such as IL-6, COX-2, PGE2, MMPs, and so on (Marcu et al., 2010; Yan et al., 2020); (Wang et al., 2018a). So, NF-κB might be a vital target for the prevention of OA.

Evodiamine (EV) exhibits excellent anti-inflammatory effects. It is a quinozole alkaloid extracted from the medicinal plant *Evodia rutaecarpa*. In previous studies, EV mitigated inflammation response *via* stimulating protective effects in an experimental model of Alzheimer’s disease (Wang et al., 2018b), kidney ischemia-reperfusion injury in rats (Eraslan et al., 2019), induced murine models of neuropathy (Wu and Chen, 2019), and colitis (Shen et al., 2019). Furthermore, it is confirmed that EV can suppress the activation of immune cells and restrain the secretion of pro-inflammatory cytokines. For example, EV downregulated the levels of IL-1β, TNF-α, and iNOS *via* NF-κB and MAPK signaling pathways in the lipopolysaccharide (LPS)-induced mastitis and adjuvant-induced arthritis, suppressing the production of TNF-α, IL-1β, and IL-6 in adjuvant-induced synovial inflammation in rats (Zhang et al., 2020; Yang et al., 2022). Hitherto, no study has demonstrated its benefits in the treatment of OA. This study is the first to investigate the protective effect of EV on OA *in vivo* and vitro. Furthermore, we aimed to demonstrate the underlying molecular mechanism of EV in the amelioration of OA. Hence, we might investigate a new effective molecule for the prevention and treatment of OA.

## Material and Methods

### Chemicals and Reagents

In this study, evodiamine (purity >98%), dimethylsulfoxide (DMSO), and type II collagenase were obtained from Solarbio (Beijing, China). Mouse IL-1β was purchased from Novoprotein (Suzhou, China). The following primary antibodies were utilized in the present study: mouse anti-collagen II, mouse anti-ADAMTS-5, mouse anti-COX-2, mouse anti-IκB, and mouse anti-GAPDH were purchased from Affinity Biosciences (Beijing, China). Mouse anti-iNOS, mouse anti-MMP-13, and mouse anti-Laminb1 were acquired from Proteintech (Wuhan, China). Primary antibodies to mouse anti-p65 were obtained from Cell Signaling Technology (Boston, MA, United States). Fetal bovine serum (FBS) and Dulbecco’s modified Eagle’s medium (DMEM)/F12 were acquired from Gibco (Grand Island, United States). And all ELISA kits were acquired from Cusabio (Wuhan, China).

### Animals

All protocols adhered to the Guide of the Animal Care and Use Committee of the Shanghai Tenth People’s Hospital. By randomly dividing, 45 two-month-old male C57BL/6 wild-type (WT) mice were selected into each group: sham group, OA group, and EV-treated OA group. As previously stated, induction of OA was performed (Moskowitz, 2006; Glasson et al., 2007; Liang et al., 2018). After using 3% pentobarbital, we induced the OA model by incising the right medial articular capsule and transecting the medial meniscotibial ligament in each mouse of the OA group and EV-treated OA group, while merely injuring the knee joints by arthrotomy in the sham control group.

### Culture of the Primary Mouse Chondrocyte

Under sterile conditions, the cartilage samples from 7-day-old mice were washed twice with PBS and cut into pieces. Under the aseptic condition, the tissues of mice articular cartilage were extracted and rinsed with PBS three times, followed by treatment with 0.15% collagenase II for another 5 h (at 37 °C) to obtain the chondrocytes. The tissue digest of mice articular cartilage was centrifuged at 1,200 rpm for 5 min, and the supernatant fluid was removed and discarded immediately. Under an atmosphere containing 5% CO_2_, the chondrocytes were suspended at 37°C in DMEM/F-12 medium (containing 10% FBS and 1% penicillin/streptomycin). The culture solution was replaced daily, and after using a 0.25% trypsin-EDTA solution, the chondrocytes were passaged when they reached nearly confluence. To reduce the loss of the phenotype, only passage 0 to 2 cells were utilized in the present study.

### Cytotoxicity of EV to Chondrocytes

The cytotoxicity of EV was evaluated for the chondrocytes by a Cell Counting kit 8 (CCK-8) assay. In short, mouse chondrocytes were incubated in 96-well plates (1 × 10^4^ cell/cm^2^) for 24 h and thereafter pretreated with various concentrations (0, 5, 10, 15, 20, 25, and 30 μM) of EV for 24 and 48 h. Afterward, each well was treated with 10 μl CCK-8 and incubated for another 2 h at 37°C. The absorbance of the solution in each well was measured at a wavelength of 450 nm using a spectrophotometer (Leica Microsystems, Germany).

### ELISAs

We used commercial ELISA kits to quantify the respective secretion of PGE2, IL-6, and TNF-α into the culture medium. In addition, we detected the NO levels in the culture medium by utilizing the Griess reaction, which was previously reported (Au et al., 2007). All experiments were performed in duplicate.

### Western Blot Analysis

The total cellular proteins were isolated using the radioimmunoprecipitation assay (RIPA) lysis buffer containing protease inhibitors and phosphatase inhibitors. The protein concentrations were determined using the BCA Protein Assay Kit (Beyotime, China) according to the manufacturer’s instructions. Then, the total proteins (20 μg) were loaded for each group and separated using 8% SDS-PAGE followed by a transfer onto the PVDF membrane. Subsequently, the membranes were blocked with 5% non-fat milk and incubated with a primary antibody (dilution 1:1,000) at 4°C overnight against iNOS, Col-II, IκBα, ADAMTS-5, MMP-13, Lamin B, p65, and GAPDH. The next day, the membranes were incubated with an HRP-conjugated secondary antibody (1:3,000) at 25°C for 2 h. Finally, the image of the protein was visualized using the Imaging System (Bio-Rad, United States) using the ECL reagent (Beyotime, China).

### Immunofluorescence Microscopy

Mouse chondrocytes were seeded onto 6-well plates with glass coverslips and incubated overnight in serum-starved medium and then merely pretreated with IL-1β (10 ng/ml) or co-stimulated with 20 μM EV and IL-1β (10 ng/ml) for 24 h. Then, the glass coverslips were rinsed with PBS three times, the chondrocytes were fixed for 30 min at 37°C with 4% paraformaldehyde, rinsed thrice with PBS, and permeabilized with 0.5% Triton X-100 for 15 min at room temperature. Then, the chondrocytes were blocked in a blocking solution (5% fetal bovine serum) for 30 min at 25°C, followed by incubation with the primary antibodies (1:100) against Col-II and p65 overnight at 4°C. The glass coverslips in the six-well plates were rinsed with PBS the next day and incubated with the fluorescein-conjugated secondary antibody (1:500) for 2 h in the dark at 25°C. Finally, the cells were incubated with DAPI for 1 min and observed under a fluorescence microscope (Leica Microsystems, Germany) after mounting the coverslip in the mountant medium followed by assessing the fluorescence intensity using ImageJ software 2.1 (Bethesda, United States).

### X-Ray

The knees of all mice were imaged with a digital X-ray system (KUB Technologies Inc.) at settings: 160 μA and 50 kV.

### Histological Analysis

The knee joints were removed from mice and subsequently fixed in 10% EDTA solution for 6 weeks after 4% paraformaldehyde fixation for 24 h. Next, with the specimens dehydrated by a series of alcohol gradients and finally embedded in paraffin wax blocks, 4-μm-thick frontal serial sections were stained with Safranin-O (S-O) and H&E to evaluate the extent of articular cartilage destruction. Thereafter, the severity of the degradation of the articular cartilage was scored according to the Osteoarthritis Research Society International (OARSI) scoring system as previously described (Pritzker et al., 2006).

### Statistical Analysis

All results of experiments, which were conducted repeatedly at least three times, are presented graphically as means ± standard deviation (SD). The original data were analyzed using SPSS 23.0 software (Chicago, IL, United States). Differences among the groups were identified by one-way analysis of variance (ANOVA) or *t*-test. *p* < 0.05 was considered to indicate statistical significance.

## Results

### Potential Cytotoxicity of EV on Mouse Chondrocytes

**FIGURE 1:**
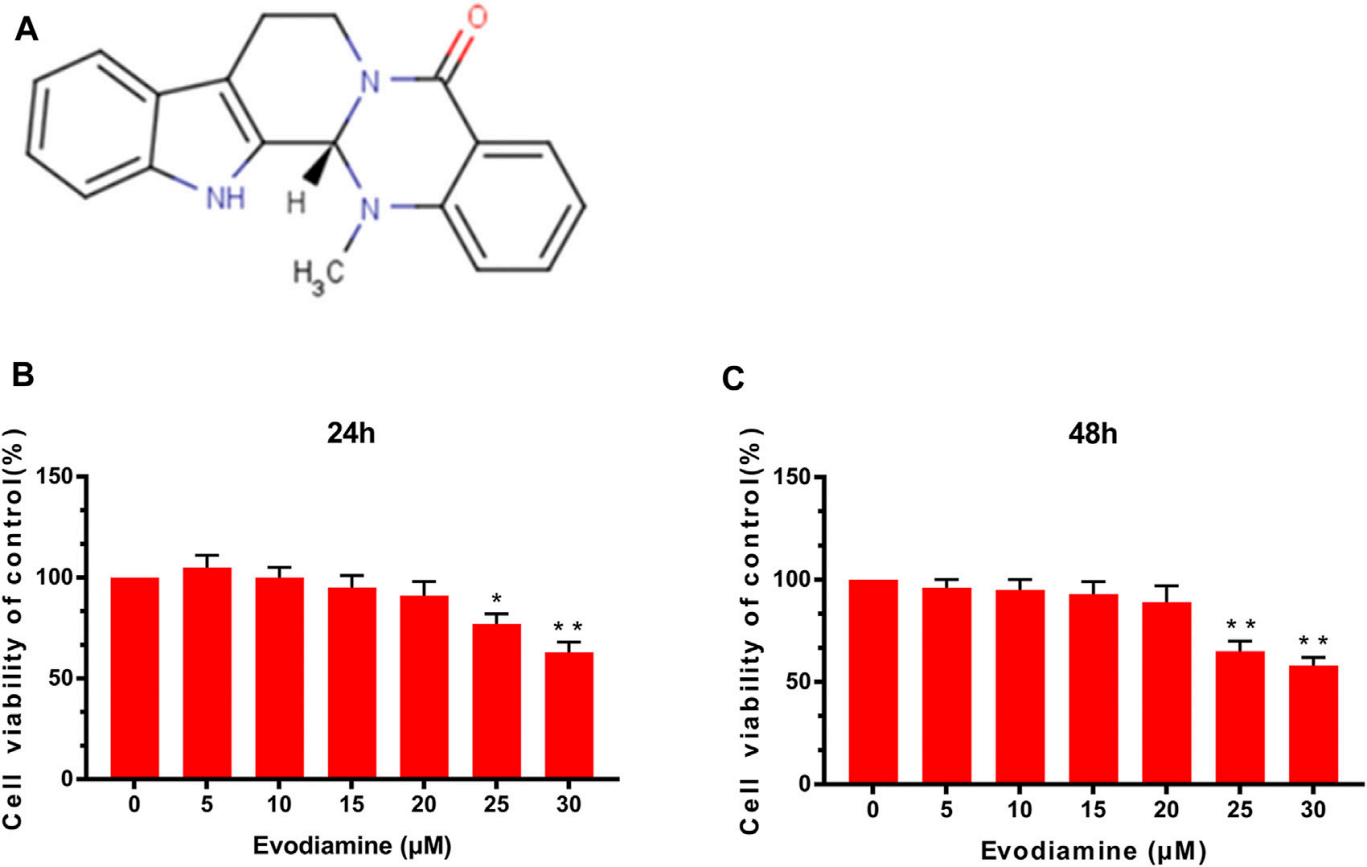
Effects of evodiamine on chondrocyte cytotoxicity. **(A)** Chemical formula of evodiamine. **(B,C)** Effect of EV on chondrocyte viability at different concentrations. Data were expressed as mean ± SD. ^∗^ *p* < 0.05, ^∗∗^ *p* < 0.01 (vs. con group, *n* = 3).

Figure 1A depicts the chemical structure of EV. The mouse chondrocytes were pretreated with various concentrations (0, 5, 10, 15, 20, 25, and 30 μM) of EV for 24 and 48 h. Afterward, using CCK-8 analysis, the effect of EV on cell viability was detected. As shown in Figures 1B,C, those concentrations of EV (0, 5, 10, and 20 μM) were chosen for subsequent experiments because no significant changes were observed in those concentrations for chondrocyte viability.

### Effect of EV on IL-6, PGE2, NO, and TNF-α Production in Chondrocytes

**FIGURE 2:**
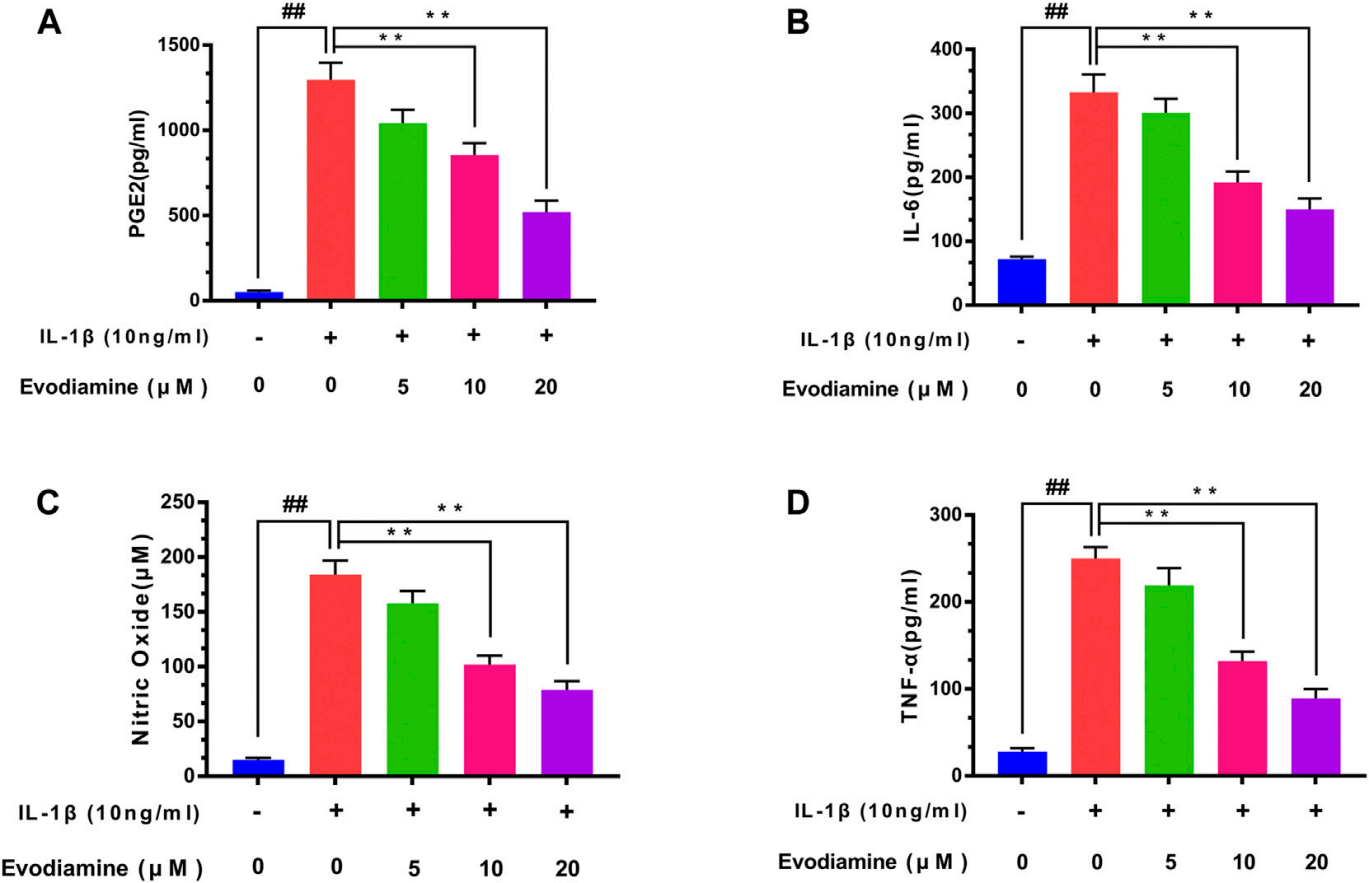
Evodiamine suppressed the production of the inflammatory factors induced by IL-1β. **(A,B,D)** Expression of PGE2, TNF-α, and IL-6 was detected by ELISA. **(C)** Nitric oxide (NO) concentrations were assessed by Griess reaction. The data were expressed as mean ± SD. Significant differences between different groups are indicated as ##*p* < 0.01, vs. control group; ^∗∗^ *p* < 0.01, vs. IL-1β alone stimulation group, *n* = 3.

ELISA was carried out to examine the content of inflammatory cytokines including TNF-α, IL-6, and PGE2 in the chondrocytes. After pretreatment with different concentrations of EV (0, 5, 10, and 20 μM) for 2 h and then with IL-1β for 24 h, the PGE2, IL-6, and TNF-α levels were assessed with ELISA kits in the culture medium and NO concentration was determined by Griess reaction. Compared to the control group, the generation of PGE2, IL-6, NO, and TNF-α upregulated obviously in IL-1β–induced chondrocytes. Interestingly, EV dose-dependently decreased the content of TNF-α, IL-6 NO, and PGE2 (Figure 2).

### Effect of EV on the Levels of COX-2 and iNOS in Mouse Chondrocytes

**FIGURE 3:**
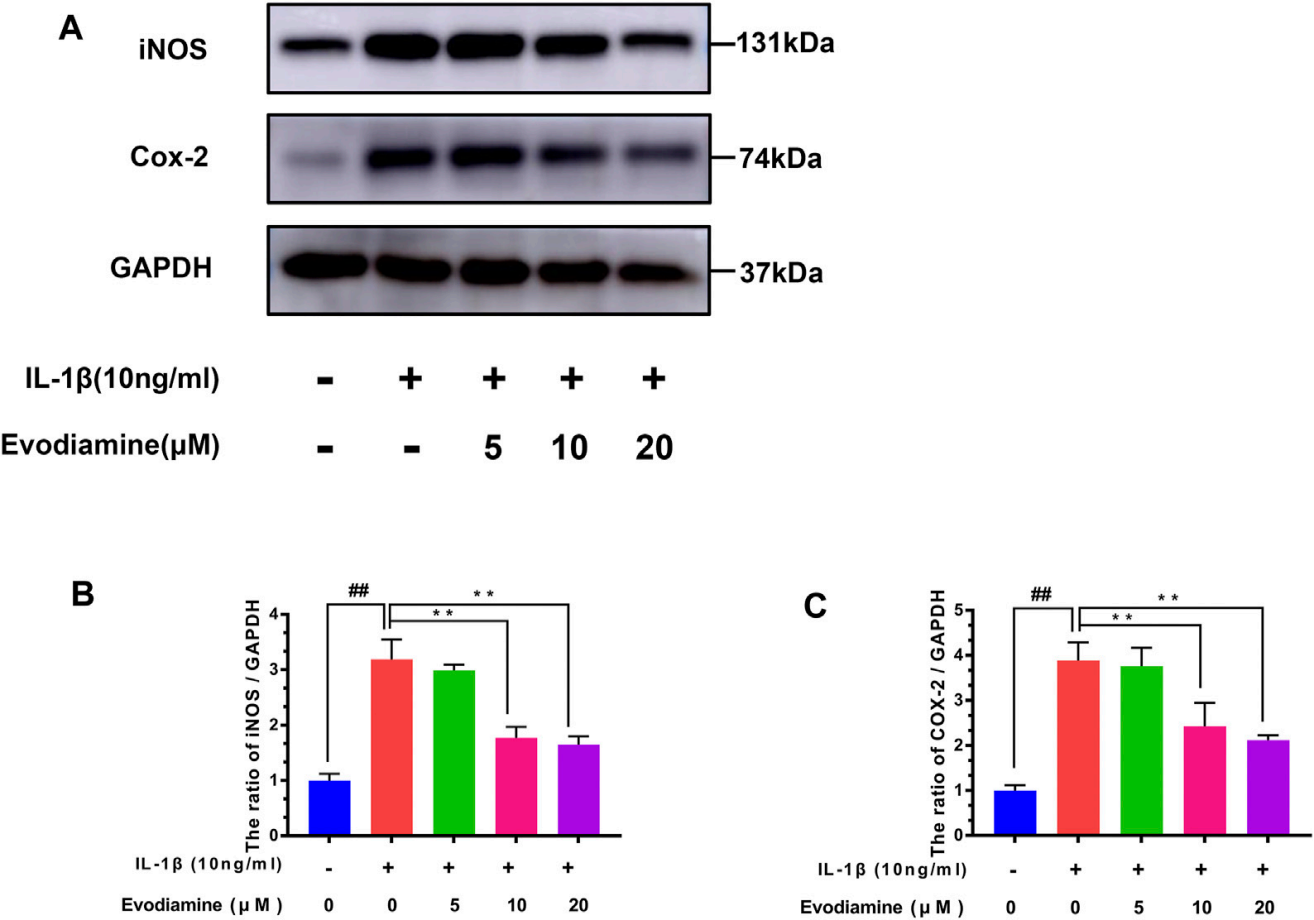
Effects of evodiamine on IL-1β–induced inflammation in mouse chondrocytes. **(A,B,C)** Level of expression of cyclooxygenase-2 (COX-2) and inducible nitric oxide synthase (iNOS) in chondrocytes of the three groups were evaluated by Western blot. The data were expressed as mean ± SD. Significant differences between different groups are indicated as ##*p* < 0.01, vs. control group; ^∗∗^ *p* < 0.01, vs. IL-1β alone stimulation group, *n* = 3.

The levels of COX-2 and iNOS were detected by Western blot. Chondrocytes were treated with EV (0, 5, 10, and 20 µM) for 24 h before the cells were stimulated with IL-1β for 2 h. IL-1β increased the content of iNOS and COX-2 protein expression (compared to the control group) (Figure 3). Interestingly, EV decreased iNOS and COX-2 protein expression of IL-1β–activated chondrocytes.

### Effect of EV on Degradation of the Extracellular Matrix in Mouse Chondrocytes

**FIGURE 4:**
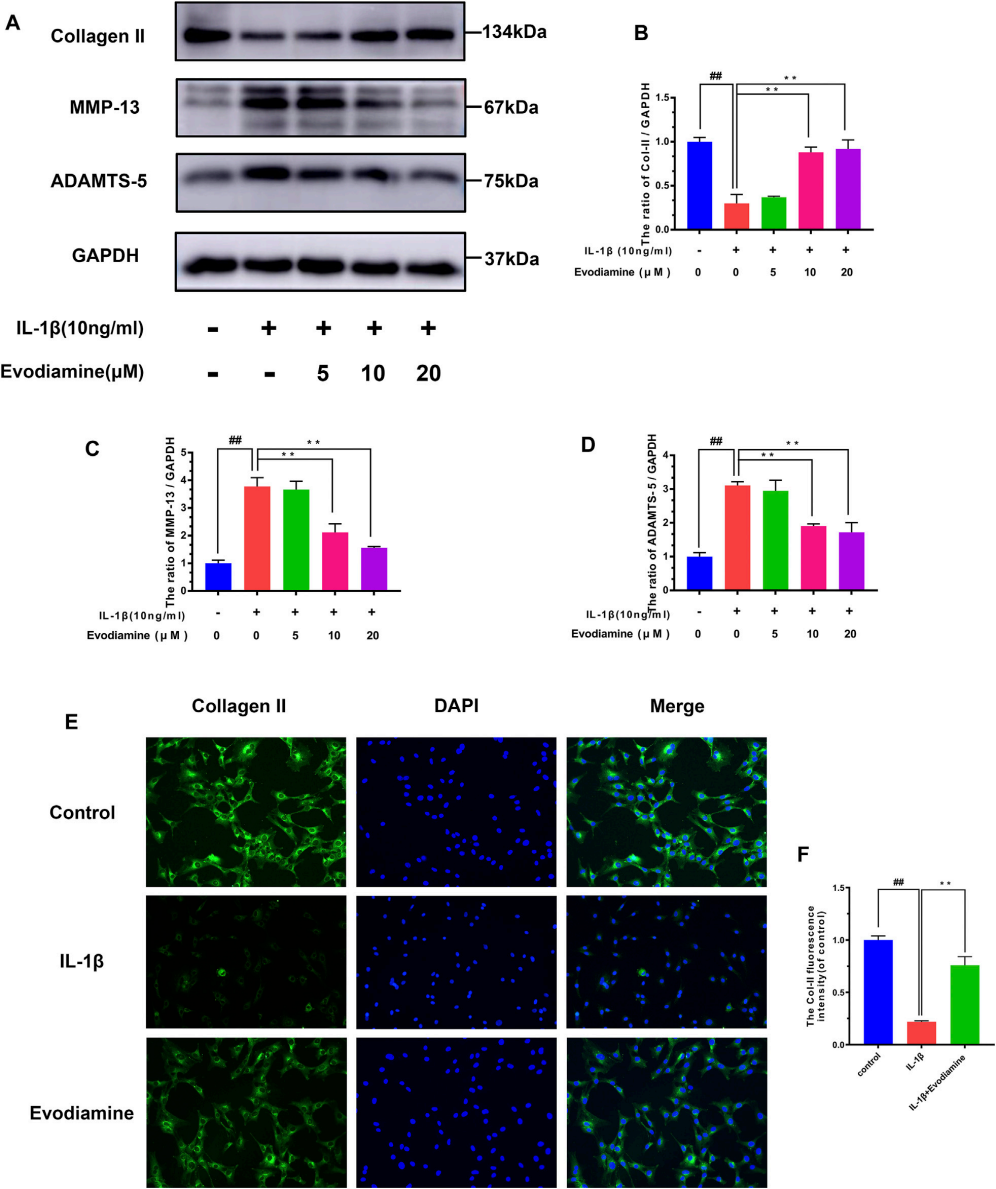
Evodiamine had a protective effect on the ECM degradation of IL-1β–induced chondrocytes. **(A,B,C,D)** Relative protein expressions of collagen-II, ADAMTS-5, and MMP-13 were quantitated by Western blot. **(E,F)** Collagen-II (green) was detected by immunofluorescence. Data were expressed as mean ± SD. Significant differences between different groups are indicated as ##*p* < 0.01, vs. control group; ^∗∗^ *p* < 0.01, vs. IL-1β alone stimulation group, *n* = 3.

Inflammatory mediators such as nitric oxide and PGE2 promote the secretion of matrix-degrading enzymes such as MMPs and ADAMTS-5. Therefore, the efficacy of EV on IL-1β–activated ECM (extracellular matrix) degradation was explored through Western blot analysis to measure the expression of MMP-13, collagen-II, and ADAMTS-5 in EV-treated mouse chondrocytes. IL-1β obviously decreased the production of collagen-II (Figures 4A–D) while increasing the production of MMP-13 and ADAMTS-5, which can degrade the ECM. However, in IL-1β–activated chondrocytes treated with EV, the production of collagen-II was noticeably upregulated, while the secretion of MMP-13 and ADAMTS-5 was downregulated in IL-1β–activated chondrocytes treated with EV. In addition to this, compared to the IL-1β group, the immunofluorescent analysis showed that treatment with EV could impressively increase the generation of Col-II in IL-1β–induced mouse chondrocytes (Figures 4E,F).

### Effects of EV on the NF-κB Signaling Pathway in IL-1β–Treated Mouse Primary Chondrocytes

**FIGURE 5:**
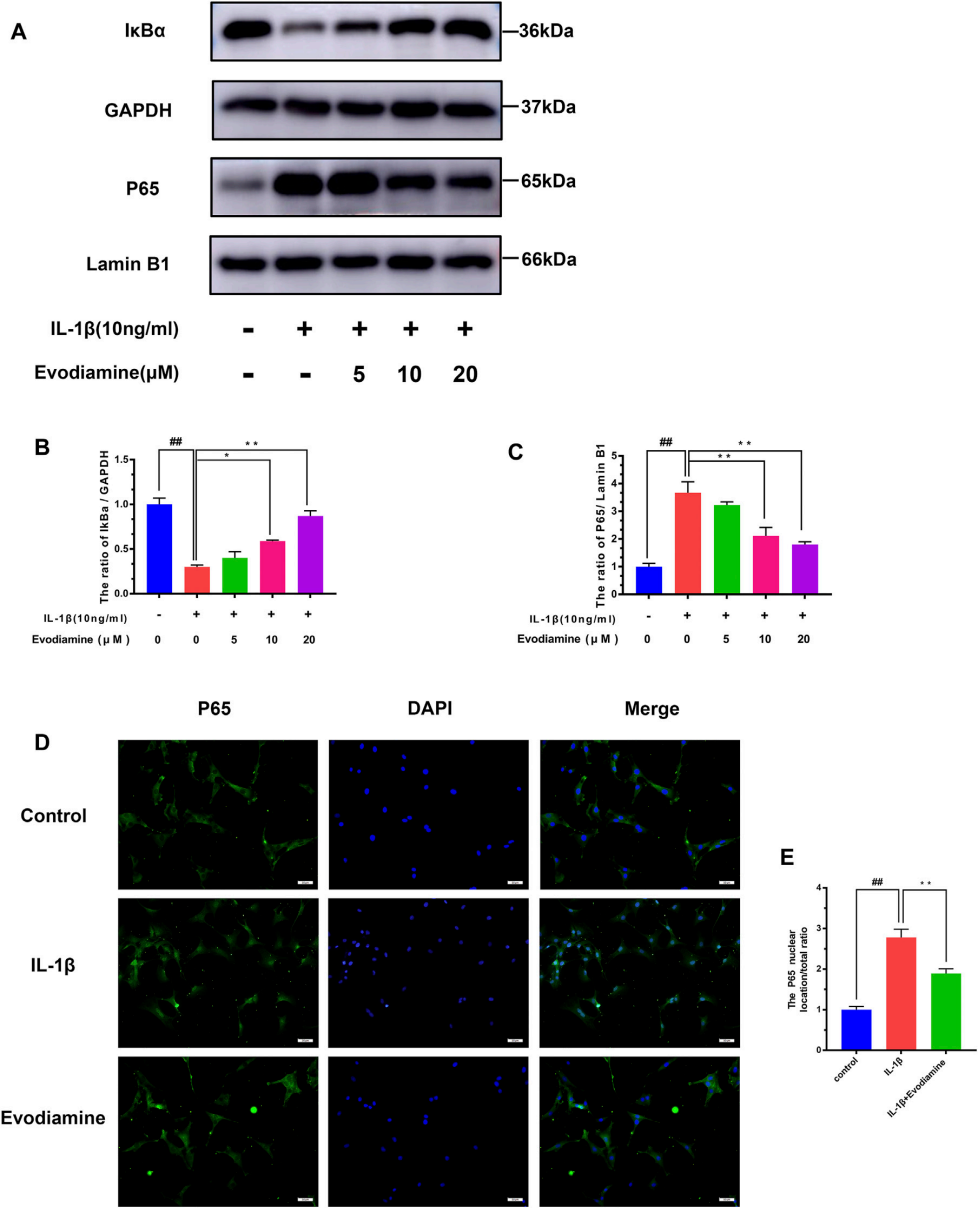
Evodiamine exerts anti-inflammatory effects in OA by modulating the NF-κB pathway. **(A,B,C)** Relative protein expression of IκB-α and p65 was quantitated using Western blot. **(D,E)** p65 (red) was detected by immunofluorescence. Data were expressed as mean ± SD. Significant differences between different groups are indicated as ##*p* < 0.01, vs. control group; ^∗∗^ *p* < 0.01, vs. IL-1β alone stimulation group, *n* = 3.

The potential molecular mechanisms of EV inhibiting the NF-κB signaling pathway were further clarified by a series of pathway activation experiments. The degradation of IκB-α was increased considerably, while noticeable phosphorylation of p65 after incubating with IL-1β for 2.5 h was observed. Nonetheless, the inhibitory effect on IL-1β–activated degradation of IκB-α and phosphorylation of p65 was discovered (Figures 5A–C). Moreover, the protective role of EV on IL-1β–stimulated NF-κB transfer to the nucleus was demonstrated by immunofluorescence microscopy (Figures 5D,E). The IL-1β group showed a distinct and intense nuclear staining for p65, while the majority of p65 localized in the cytoplasm in the control group, implying the NF-κB subunit transferred into the nuclear after being stimulated with IL-1β. Interestingly, the IL-1β–activated nuclear translocation of p65 subunits decreased obviously, which indicates that nuclear translocation of NF-κB p65 could be inhibited by EV.

### EV Alleviates Progression of OA in a Mouse Model

**FIGURE 6:**
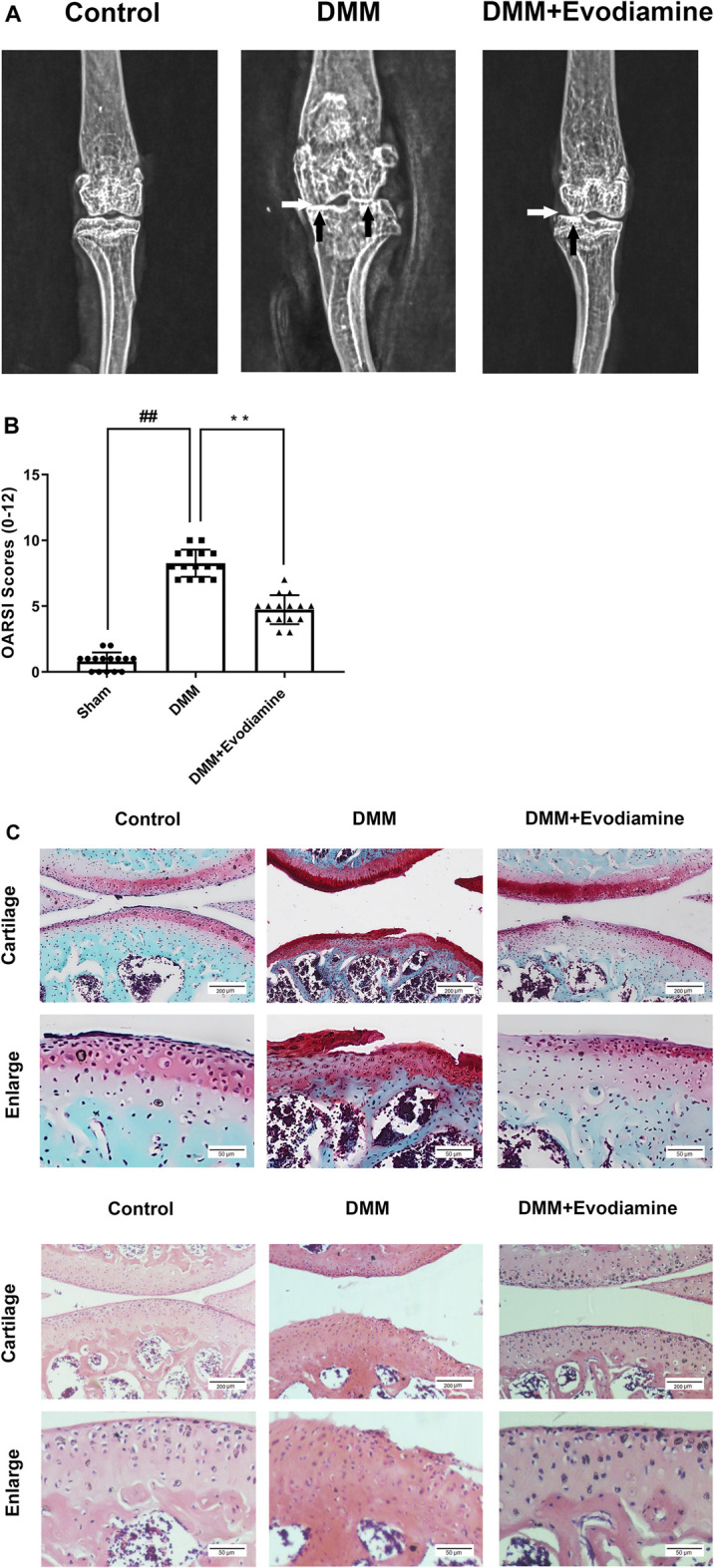
(Continued).

To investigate the potential role of EV in OA, EV (10 mg/kg) was injected intra-articularly and after 4 weeks post-surgery to establish the mouse OA model. Then, the knee joints were evaluated with S-O staining of histological sections and X-ray images. Compared to the sham group, the OA model was successfully established in the DMM group with the disappearance of the joint space, calcification, and the presence of osteophytes (Figure 6A). However, the severity of OA was reduced in the DMM + EV group in comparison to the DMM group. Meanwhile, in S-O staining and H&E staining, severe cartilage erosion and depletion of proteoglycans were observed in the DMM group and alleviated in the DMM + EV group (Figure 6C). Furthermore, the change of OARSI scores was consistent with the change in former S-O staining, which significantly increased in the DMM group and then significantly decreased in the DMM + EV group (Figure 6B).

## Discussion

Because of the significant aging of the population globally, osteoarthritis has been the most frequent musculoskeletal disease. We still lack an effective strategy for the conservative treatment of OA. Most patients still need total knee arthroplasty. Because of their high cost, they place a heavy burden on the healthcare system and challenge future therapeutic applications. Hitherto, NSAIDs are the most popularly applied drugs for osteoarthritis treatment. However, they merely relieve the symptoms and cannot stop the progression of the OA. Meanwhile, patients with long-term use of NSAIDs would suffer various severe side effects. So, it is necessary and imperative to explore a safer and more effective drug. In this study, to demonstrate the chondro-protective of EV in OA, we utilized IL-1β–stimulated mouse chondrocytes *in vitro* and the DMM model *in vivo*. Consistent with the aforementioned result, EV therapy remarkably suppressed the production of inflammatory cytokines and the degradation of Col-II and significantly ameliorated cartilage degeneration and OARSI scores. Therefore, EV possesses good antiarthritic activity.

IL-1β plays a vital role in the deterioration of OA by stimulating the expression of inflammatory mediators (Daheshia and Yao, 2008; Jenei-Lanzl et al., 2019). In chondrocytes, IL-1β can upregulate the expression of COX-2 and iNOS and the production of NO, PGE2, and TNF-α (Daheshia and Yao, 2008; Wu et al., 2020). Then, PGE2 and NO could promote the deterioration of OA by stimulating the expression of MMPs and inhibiting the production of macromolecular substances (Kapoor et al., 2011). Surprisingly, in our present study, our results showed that EV could decrease the production of COX-2 and iNOS and inhibit the overproduction of IL-6, NO, and PGE2.

MMPs have been confirmed to be associated with the regulation of inflammatory response and degradation of the ECM (Tetlow et al., 2001; Pacheco-Fernandez et al., 2020). In particular, the MMP-13 plays a key role in OA by deteriorating the degradation of proteoglycans and collagen-II, which are the major components of the ECM (Neuhold et al., 2001; Pasternak and Aspenberg, 2009). In addition, ADAMTS-5, a member of the ADAMTS family of proteins, plays a pivotal role in the progression of OA *via* cleavage of aggrecan or degradation of the ECM (Verma and Dalal, 2011; Pérez-García et al., 2016). It has been investigated that a humanized ADAMTS-5 inhibitor can effectively prevent the progression of OA (Chu et al., 2013). In this study, the result of the experiment showed that EV significantly suppressed IL-1β–stimulated MMP-13 and ADAMTS-5 protein expression and increased the production of Col-II in mouse chondrocytes.

Increasing pieces of evidence indicate that the NF-κB pathway is widely involved in the triggering and development of OA by regulating pro-inflammatory factors (Marcu et al., 2010; Lepetsos et al., 2019). Under normal conditions, NF-κB dimers are located in the cytoplasm as an inactive form bound with IκBα molecules. After IκBα degradation was stimulated by diverse inflammatory signals, the expression of all kinds of inflammation-related proteins was upregulated after NF-κB hetero-dimers translocated into the cell nucleus (Rigoglou and Papavassiliou, 2013). The use of specific NF-κB inhibitors has been reported to alleviate the aggravation of IL-1β–induced osteoarthritis in mice and rats (Pan et al., 2017; He et al., 2021). Increasing evidence also shows PI3K/Akt, an intracellular signaling pathway, can regulate its upstream NF-κB pathway (Chen et al., 2013). The downstream inflammatory factors such as PGE2, iNOS, and COX-2, MMPs in cells would be increased after the activation of the NF-κB pathway (Jimi et al., 2019; Feng et al., 2020; Wu et al., 2020). In the present study, the immunofluorescence results showed that EV pretreatment could inhibit the translocation of NF-κB hetero-dimers into the nucleus. These results suggest that it is an underlying pathway for EV to exert chondro-protective effects on cells *via* the NF-κB signaling pathway.

Previous studies clarified that the DMM surgical instability mouse model of OA, similar to human OA, has sufficient sensitivity to be utilized in OA research as the first choice (Glasson et al., 2007). Pathological changes such as the disappearance of the joint space, calcification, and the presence of osteophytes occurred in the DMM model. Meanwhile, OA-related structural damage was significantly mitigated after EV administration in the DMM + EV group. Our results demonstrated EV to possibly inhibit the activation of the NF-κB pathway to resist intra-articular inflammation and cartilage degeneration in OA progression. However, the NF-κB pathway can also regulate autophagy and apoptosis in IL-1β–induced chondrocytes. Whether EV plays a chondro-protective effect on IL-1β–induced chondrocytes by regulating autophagy and apoptosis needs to be investigated in our further study.

## Conclusion

We investigated the molecular mechanism by which EV can inhibit IL-1β–stimulated inflammation and ECM degradation in mouse chondrocytes through NF-κB pathways *in vitro*. Furthermore, *in vivo*, the OARSI scores could be decreased in surgically induced OA models. In addition, these findings imply that EV could be a potential therapeutic candidate for the treatment of OA.

## Data Availability

The original contributions presented in the study are included in the article/Supplementary Material; further inquiries can be directed to the corresponding authors.
